# Failure to activate the in-hospital emergency team: causes and outcomes

**DOI:** 10.5935/0103-507X.20160075

**Published:** 2016

**Authors:** Vera Barbosa, Ernestina Gomes, Senio Vaz, Gustavo Azevedo, Gonçalo Fernandes, Amélia Ferreira, Rui Araujo

**Affiliations:** 1 Hospital Pedro Hispano, Unidade Local de Saúde de Matosinhos, EPE - Senhora da Hora, Portugal.

**Keywords:** Hospital rapid response team, Afferent pathways, Hospital mortality, Equipe de respostas rápidas de hospitais, Vias aferentes, Mortalidade hospitalar

## Abstract

**Objective:**

To determine the incidence of afferent limb failure of the in-hospital Medical Emergency Team, characterizing it and comparing the mortality between the population experiencing afferent limb failure and the population not experiencing afferent limb failure.

**Methods:**

A total of 478 activations of the Medical Emergency Team of *Hospital Pedro Hispano* occurred from January 2013 to July 2015. A sample of 285 activations was obtained after excluding incomplete records and activations for patients with less than 6 hours of hospitalization. The sample was divided into two groups: the group experiencing afferent limb failure and the group not experiencing afferent limb failure of the Medical Emergency Team. Both populations were characterized and compared. Statistical significance was set at p ≤ 0.05.

**Result:**

Afferent limb failure was observed in 22.1% of activations. The causal analysis revealed significant differences in Medical Emergency Team activation criteria (p = 0.003) in the group experiencing afferent limb failure, with higher rates of Medical Emergency Team activation for cardiac arrest and cardiovascular dysfunction. Regarding patient outcomes, the group experiencing afferent limb failure had higher immediate mortality rates and higher mortality rates at hospital discharge, with no significant differences. No significant differences were found for the other parameters.

**Conclusion:**

The incidence of cardiac arrest and the mortality rate were higher in patients experiencing failure of the afferent limb of the Medical Emergency Team. This study highlights the need for health units to invest in the training of all healthcare professionals regarding the Medical Emergency Team activation criteria and emergency medical response system operations.

## Abstract (pt)

**Objetivo:**

Determinar a incidência de falha na ativação da via aferente da Equipe de Emergência Médica intra-hospitalar, caraterizando-a e comparando a mortalidade dessa população com a da população em que não se verificou falha na ativação da via aferente.

**Métodos:**

Entre janeiro de 2013 e julho de 2015, ocorreram 478 ativações da Equipe de Emergência Médica do Hospital Pedro Hispano. Após a exclusão de registos incompletos e ativações para doentes com menos de 6 horas de internamento hospitalar, obtivemos uma amostra de 285 ativações. A amostra foi dividida em dois grupos: o grupo com falha na ativação da via aferente e o grupo em que não ocorreu falha na ativação da via aferente da Equipe de Emergência Médica. As duas populações foram caracterizadas e comparadas. A significância estatística foi considerada para p ≤ 0,05.

**Resultado:**

Em 22,1% das ativações, verificou-se falha na ativação da via aferente. Relativamente ao estudo causal, verificamos existir diferença estatisticamente significativa quanto aos critérios de ativação da Equipe de Emergência Médica (p = 0,003) no grupo com falha na ativação da via aferente, encontrando taxa mais elevada de ativação da Equipe de Emergência Médica por paragem cardiorrespiratória e disfunção cardiovascular. Em relação às consequências, no grupo em que ocorreu falha na ativação da via aferente houve uma maior taxa de mortalidade imediata e à data de alta hospitalar, sem significado estatístico. Não encontramos diferenças significativas com relação aos outros parâmetros.

**Conclusão:**

Nos doentes em que houve falha da ativação da via aferente da Equipe de Emergência Médica, a incidência de paragem cardiorrespiratória e a taxa de mortalidade foram maiores. Este estudo reforça a necessidade de as unidades de saúde investirem na formação de todos os profissionais de saúde sobre os critérios de ativação da Equipe de Emergência Médica e o funcionamento do sistema de resposta a emergência médica.

## INTRODUCTION

In-hospital cardiac arrest (IHCA) occurs in up to 5:1,000 adult hospitalizations.^(1)^ Despite post-resuscitation interventions, IHCA is usually associated with a worse prognosis than that of out-of-hospital cardiac arrest (CA).^(2)^ Furthermore, approximately 50% of these hospitalizations are potentially preventable^(3)^ because progressive clinical deterioration usually occurs.^(4,5)^

Several hospital units have developed medical emergency response systems (MERS) for preventing CA by identifying and correcting factors in patients at risk for IHCA in a timely manner.^(6-8)^ These systems have afferent and efferent limbs, regardless of the model. The afferent limb consists of clinical monitoring and surveillance, the detection of patient clinical deterioration, the knowledge of the Medical Emergency Team (MET) activation criteria and its activation. The MET is the efferent limb. The healthcare professionals at each institution should be aware of and familiar with the MET activation criteria in force in their hospital. Despite the investment in training in this area, the literature recognizes the existence of deficient levels of knowledge and the persistence of barriers, which result in delayed MET activation. The MET is not activated in 30 to 78% patients with MET activation criteria; even when activation occurs, there is an average delay of 16 hours.^(9-13)^ This afferent limb failure (ALF) has a negative impact on prognosis^(14)^ and results in obvious expenses for the health system.

The MERS of *Hospital Pedro Hispano* has been in operation since 1998. Until 2002, the activation criteria were exclusively CA or respiratory arrest. From 2002, the criteria were broadened, and teams experienced in the emergency care and management of seriously ill patients are now activated using predefined criteria.^(15)^ Based on the records of MET activations at *Hospital Pedro Hispano*, we aimed to determine the incidence of in-hospital MET afferent limb failure, characterizing it and comparing the mortality of the population experiencing ALF with that of the population not experiencing ALF.

## METHODS

The study was performed at *Hospital Pedro Hispano, Unidade Local de Matosinhos* (ULSM), E.P.E., which serves the municipality of Matosinhos and its 175,478 inhabitants. This hospital had 345 beds distributed according to different medical specialties (internal medicine, cardiology, general surgery, ophthalmology, otorhinolaryngology, gynecology, obstetrics, pediatrics, urology, orthopedics, the intensive care unit [ICU] and the multidisciplinary intermediate care unit [MICU]).^(16)^*Hospital Pedro Hispano* received patients referred from the *Centro Hospitalar da Póvoa de Varzim/Vila do Conde*, which has a capacity of 145 beds serving a population of 143,000 inhabitants.^(17)^

Data were collected from the internal emergency forms, which were available in the electronic medical records and were filled out by the MET physician at the end of each activation. Some incomplete forms were filled out when possible to gather the missing data using the records of vital parameters, recorded by the nurses of the SClínico^®^ System (Ministry of Health, Government of Portugal, Lisbon, Portugal).

All MET activations from January 2013 to July 2015 were analyzed, totaling 478. Activations for outpatients (n = 29) and patients hospitalized for less than 6 hours (n = 48) and activations with incomplete forms (n = 116) were excluded. The final sample consisted of 285 activations.

The MERS afferent limb is based in the ICU, which, once activated, sends a team trained in emergency response and the management of seriously ill patients. The afferent limb is composed of healthcare professionals working in wards and trained in Basic Life Support (BLS) and MET activation criteria identification. A training program with regular recertification in BLS, Immediate Life Support (ILS) and Advanced Life Support (ALS) for target populations selected based on the care responsibilities of the professionals is also included in this model. The training program is held at an Emergency School of the ULSM, accredited by the Portuguese Resuscitation Council. The MERS includes emergency carts with standardized content that are distributed at selected sites in the hospital to support responses to emergency situations. The MERS is preferentially contacted by dialing a direct telephone number and includes an audit program for system maintenance quality, with designated managers for system-wide management.

The MET consists of a physician and a nurse, trained in ALS and experienced in resuscitation, who predominantly work in the ICU. The MET is available 24 hours a day and is activated when specific criteria are met by dialing a direct number (2211) to call the team at the ICU.

The existence of a MET activation delay longer than 15 minutes, in the presence of the activation criteria, was considered an ALF. This time period was measured using the MET activation time and the time of the measurement of vital signs or clinical records meeting the activation criteria. The sample was divided into two groups: the MET ALF group and the MET non-ALF group. The activation criteria are outlined in table 1.

**Table 1:** Medical Emergency Team activation criteria

Airway	Threatened airway
Breathing	Respiratory arrest
	Respiratory rate < 5 or > 36 cycles/minute
Circulation	Heart rate < 40 or > 140bpm
	Systolic blood pressure < 90mmHg
	Cardiac arrest
Neurological	Sudden consciousness disturbance
	Decrease in Glasgow Coma Scale score ≥ 2 points
	Repeated convulsive seizures
Other	Any patient with a concerning clinical status who does not meet the above criteria

### Statistical analysis

Statistical analysis was performed using the program Statistical Package for the Social Sciences (SPSS), version 17.0 (SPSS Inc., Chicago, Illinois, United States). The statistical description was performed using the counts and percentages for categorical variables and the median, maximum and minimum values for continuous variables. Comparisons between samples were performed using the chi-squared test. We considered the results to be statistically significant when p < 0.05.

## RESULTS

A total of 478 MET activations occurred at *Hospital Pedro Hispano* from January 2013 to July 2015. The final sample is outlined in table 2.

**Table 2:** Characterization of the study population

Variables	
Number of activations	285
Mean age (years)	75 (11 - 94)
Females	162 (56.8)
Activation site	
Surgery	51 (17.1)
Gynecology	7 (2.6)
Imaging	12 (4.3)
Internal Medicine	123 (43.3)
Ophthalmology	3 (1.2)
Orthopedics	52 (18.3)
Otorhinolaryngology	8 (2.9)
Pediatrics	1 (0.4)
Urology	28 (9.9)
Limitation to MET intervention	
Documented DNR order	12 (4.2)
Documented treatment limitation decision	31 (10.9)
Previous hospitalization in ICU, MICU or ER	16 (5.6)
Previous invasive procedures	
Cardiac catheterization	2 (0.7)
Surgical intervention	29 (10.2)
MET activation criteria	
Altered state of consciousness	113 (43.2)
Convulsive seizures	6 (2.1)
HR < 40 or >140bpm	53 (18.6)
RR < 5 or > 35 cycles/minute	44 (15.4)
Respiratory arrest	31 (10.9)
SBP ≤ 90mmHg	41 (14.4)
CA	94 (33.0)
Threatened airway	55 (19.2)
Activation criteria present 6 hours before	63 (22.1)
DNR order after MET	16 (5.6)
Treatment limitation decision after MET	25 (8.8)
Mortality	
At the end of the activation	81 (28.4)
At hospital discharge	46 (51.2)

MET - Medical Emergency Team; DNR - do not resuscitate; ICU - intensive care unit; MICU - multidisciplinary intermediate care unit; EE - emergency room; HR - heart rate; RR - respiratory rate; SBP - systolic blood pressure; CA - cardiac arrest. Values are expressed as median numbers (%).

Of a total of 285 activations, 58.6% were for female patients. The patient ages ranged from 11 to 94 years, with a median of 75 years. Most activations occurred for patients older than 75 years (55.1%). Regarding the MET activation site, most (52.3%) activations occurred for patients hospitalized in surgical wards (general surgery, otorhinolaryngology, ophthalmology, gynecology, orthopedics and urology), compared with 43.5% of cases hospitalized in medical wards (internal medicine and pediatrics). In 4.2% of cases, the patients were in the imaging unit. Of the 285 activations, 10.9% were for patients who had invasive procedures performed in the previous 24 hours (0.7% were cardiac catheterizations, and 10.2% were surgical procedures); in 5.6% of cases, the patients had been in the ICU, the MICU or the emergency room in the previous 24 hours.

The most common MET activation criterion was an altered state of consciousness (43.2%), followed by CA (33.0%) and a threatened airway (19.2%). Treatment limitation decisions occurred in 10.9% of cases, and do-not-resuscitate (DNR) orders were documented in 4.2% of cases. At the end of the MET intervention, 28.4% of deaths were recorded; DNR orders were followed in 5.6% of cases, and treatment limitation decisions were noted in 8.8% of cases. The overall hospital mortality was 51.2%. Multiple MET activations in the same hospitalization episode occurred in nine patients (3.15%).

Afferent limb failure was observed in 22.1% of activations. Table 3 outlines the comparison between study groups. We found a significant difference of the MET activation criteria. The ALF group had a higher MET activation rate for CA and heart rates (HRs) < 40 or > 140bpm and/or systolic blood pressure (SBP) < 90mmHg than the non-ALF group. The ALF group also had higher immediate mortality rates and higher mortality rates at hospital discharge than the non-ALF group, but the differences were non-significant. No significant differences in other parameters were found.

**Table 3:** Comparison of the two study groups

	All activations	Activations without ALF	Activations with ALF	p value
Number of activations	285	222 (77.9)	63 (22.1)	
Age (years)				0.142
< 65	67 (23.5)	52 (23.4)	15 (23.8)	
65 - 75	61 (21.4)	53 (23.9)	8 (12.7)	
> 75	157 (55.1)	117 (52.7)	40 (63.5)	
Sex (females)	162 (56.8)	123 (55.4)	39 (61.9)	0.358
Activation site				0.487
Surgery	149 (52.3)	116 (52.3)	33 (52.4)	
Internal Medicine	124 (43.5)	95 (42.8)	29 (46.0)	
Imaging	12 (4.2)	11 (5.0)	1 (1.6)	
DNR order	12 (4.2)	9 (4.1)	3 (4.8)	0.805
Treatment limitation decision	31 (10.9)	22 (9.90)	9 (14.3)	0.540
Prior invasive procedures	31 (10.9)	23 (10.4)	8 (12.7)	0.599
MET activation criteria				0.003
Compromised airway	10 (3.5)	6 (2.7)	4 (6.3)	
RR < 5 or > 35 cycles/minute	45 (15.8)	41 (18.5)	4 (6.34)	
HR < 40; > 140bpm or SBP ≤ 90	75 (26.3)	50 (22.5)	25 (39.7)	
Altered state of consciousness	66 (23.2)	58 (26.1)	8 (12.7)	
CA	85 (29.8)	65 (29.3)	20 (31.7)	
Others	4 (1.4%)	2 (0.9)	2 (3.2)	
DNR order after MET	16 (5.6)	12 (5.40)	4 (6.35)	0.774
Treatment limitation decision after MET	25 (8.8)	16 (7.25)	9 (14.3)	0.080
Mortality				
At the end of the activation	81 (28.4)	60 (27.0)	21 (33.3)	0.327
At hospital discharge	146 (51.2)	112 (50.5)	34 (54.0)	0.622

ALF - afferent limb failure; DNR - do not resuscitate; MET - Medical Emergency Team; RR - respiratory rate; HR - heart rate; CA - cardiac arrest; SBP - systolic blood pressure. Values are expressed as numbers (%).

## DISCUSSION

This cross-sectional retrospective study evaluated data for patients treated by the MET. One in five activations were triggered by situations whose subsequent evaluations identified the existence of MET activation criteria in the previous 6 hours, thus indicating MET ALF, which resulted in an increased number of activations for CA and increased mortality.

This problem has already been identified in the literature in the study by Trinkle et al.,^(9)^ who found ALF, defined as the presence of MET activation criteria from 15 minutes to 24 hours before MET activation, CA or ICU admission. The prevalence of ALF was 22.8% among the 575 events recorded. The number of ALF cases identified in our study was similar (22.1%), and this high incidence justifies a reflection aimed at identifying possible causes to be corrected and starting urgent corrective interventions.

In our study, we observed that higher percentages of cases of CA and cardiovascular dysfunction and higher immediate mortality rates and higher mortality rates at hospital discharge were observed for patients in the ALF group than for patients in the non-ALF group, but the differences were non-significant. These results are corroborated in the literature. An observational prospective study by Tirkkonen et al.^(14)^ reported that ALF is independently associated with an increase in hospital mortality.

At this point, we must analyze several factors, including MET maturity and the training of healthcare professionals working in wards and hospital units with different levels of patient monitoring, including ICUs, intermediate care units and the operating room. Situations of DNR orders and treatment limitation decisions, albeit not explicitly assumed and which may cause inappropriate MET activations, must also be analyzed. Another factor that may have contributed to this high ALF rate is the sensitivity of the activation criteria used.

The studied MET characteristics are similar to those reported for other METs. Calzavacca et al.^(11)^ report that the MET of Melbourne Hospital in Australia also consists of a physician and a nurse specifically trained in ALS and resuscitation. The MET operates 24 hours a day, and electronic medical records are completed for all activations at the end of each activation, similar to our MERS. The MET may be activated for any person in the presence of MET activation criteria. Calzavacca et al. compared two cohorts of patients with similar characteristics: a recent cohort receiving a MET review and another cohort receiving a MET review 5 years earlier at the start of its implementation, concluding that decreased ALF was observed in the group with the 5-year team and explaining that team maturation leads to decreased ALF.^(11)^

The MET analyzed in this study has existed in the hospital since 1998. From 1998 to 2002, the MET activation criteria were exclusively CA or respiratory arrest; the criteria have been extended since 2002. A study conducted in our hospital included all MET activations for patients hospitalized from 2002 to 2006 and found a decrease in CA correlated with the increase in MET maturity, combined with an integrated training program for healthcare professionals.^(18)^ Thus, at this point in time, we may assume that the team and the entire system are presently mature and that no AFL would occur.

In hospitals, new professionals are constantly hired, including for nursing teams. The new staff members may not be adequately informed and familiar with the MET criteria and method of activation or may still fear performing an unnecessary activation, causing an increase in MET ALF. Several studies suggest that increasing and improving training, familiarization, the continuous training of healthcare professionals and the existence of audits and feedback of METs are essential for MET success and ALF reduction.^(14,19,20)^ There is a need to strengthen the knowledge of MET activation criteria for those who assess and register patient monitoring parameters by implementing training sessions and presenting the data shown in this study. In the specific case of our in-hospital emergency system, training in BLS has decreased due to budget constraints, which may be correlated with this increase in ALF among ALS teams.

Another factor that may have contributed to the ALF values observed is that patients had been previously admitted to different hospital units, including the operating room, the ICU, the MICU and the emergency room, during the same hospitalization for 5.6% of the MET activations. Early discharges from those hospital units may have occurred. Therefore, greater investment towards increasing the sensitivity and knowledge of healthcare professionals regarding procedures and pathologies requiring longer hospitalizations in different units, with higher levels of clinical surveillance and monitoring, should be considered.

The level of patient monitoring depends on the hospital unit in which patients are hospitalized. In our study, most activations occurred for wards in which monitoring is performed with standardized assessments of vital signs every 6 hours and according to the recommendations from the Rapid Response Team Consensus Conference, which advocates that vital signs should be assessed every 6 to 12 hours.^(21)^ However, continuous monitoring is associated with a better outcome in patients with prior IHCA^(2,22)^ because less frequent monitoring may delay the detection of instability and may contribute to increased MET ALF. Other studies do not support this premise, including one study reporting that 40.6% of anomalies found in continuous monitoring resulted from artifacts.^(23)^

Another factor that may have contributed to bias in the MET ALF rate found in our study is the existence of activations for patients with prior DNR orders (4.2%) and treatment limitation decisions (10.9%). In these cases, tolerance and delayed MET activation occur more frequently in the presence of abnormal vital signs. The percentage of cases found in our study was similar to that of cases described by the MERIT multicenter study (7.85%).^(24)^

In addition to cases with previously documented DNR orders, it should be noted that there could be cases of DNR orders that were not documented or conveyed to the nursing team of the ward or the MET. Mitchell et al.^(25)^ reported that DNR orders existed in 6.2% of cases but were not documented or had not been conveyed to the MET. The DNR/treatment limitation process requires time to communicate with the patient and relatives and among clinicians involved in treating the patient. MET activation may be a valuable opportunity to start this process.^(25)^ In the case of our study, higher rates of DNR orders (6.35%) and treatment limitation decisions (14.3%) were found in the group of ALF patients than in the group of non-ALF patients at the end of the MET intervention.

Strategies rendering DNR orders and their documentation and communication to the other elements of the team more effective must be developed.

Another factor that may have contributed to this high MET ALF rate is the use of relatively few sensitive activation criteria in our system. In-hospital emergency systems using more sensitive criteria, including the National Early Warning System (NEWS) and the Modified Early Warning System (MEWS), which use scoring and alert systems based on a set of criteria, including temperature and oxygen saturation, in addition to urinary output, have been reported in the literature.^(26,27)^

### Study limitations

This study was conducted in only one center, and the results may not apply to other settings. Furthermore, the analysis of the activation criteria present 6 hours before the actual MET activation had some incomplete records. Thus, those patients were not included the analysis, which may have led to bias in the sample.

## CONCLUSION

The timely identification of patients with clinical deterioration does not always occur in hospital units. An increased incidence of cardiac arrest and higher mortality rates are observed in patients for whom this identification fails.

This study showed the need for hospital units to invest in training and familiarizing all health professionals with the Medical Emergency Team activation criteria and in-hospital emergency system operations.
